# Subnutrition during gestation and lactation affects testicular development, serum testosterone concentration and androgen receptor expression in myoid cells of mature rats

**DOI:** 10.1590/1984-3143-AR2025-0119

**Published:** 2026-05-25

**Authors:** Patricia Genovese, Emiliano Herrera, Martín Duque, Andrea Fernandez, Alejandro Bielli

**Affiliations:** 1 Unidad Académica de Histología y Embriología, Departamento de Biociencias Veterinarias, Facultad de Veterinaria, Universidad de la República, Montevideo, Uruguay; 2 Unidad Académica de Endocrinología y Metabolismo Animal, Imagenología y Análisis clínicos, Departamento de Clínica y Hospital Veterinario, Facultad de Veterinaria, Universidad de la República, Montevideo, Uruguay

**Keywords:** androgen receptor, lactation, testes, fetal programming

## Abstract

This study aimed to investigate one- year- old male rat sensitivity to fetal programming effects of subnutrition during gestation and lactation. Thirty primiparous female 3 month old Wistar rats were all mated by the same male. Pregnant females were organised in two experimental groups: control (CG n= 15; *ad libitum* food access) and restricted group (RG n= 15; food intake 50% of the control group). Morphometric and immunohistochemical techniques were employed to determine the number of Sertoli cells and both the distribution and relative abundance of androgen receptor in Sertoli, Leydig, and myoid cells. Additionally, the proportions of proliferating and apoptotic cells were analyzed. Testosterone concentration was also measured in blood serum and determined by a direct solid-phase radioimmunoassay. Our findings indicate that nutritionally restricted animals at early developmental stages exhibited reduced body weight (CG 592,9±33,4*** vs RG 527,5±23,5), testicular weight (CG 1,82±0,05*** vs RG 1,69±0,06), and body length (CG 47,2±0,7*** vs RG 45,1±1,6). We also observed in the same experimental group a lower number of Sertoli cells both per cross section of the seminiferous tubule (CG 23,4±0,32*** vs RG 21,8±0,34) and total per testicle (CG 134,6±24,9 x 10^4^* vs RG 111,4±14,4 x 10^4^). We found no treatment effect on the volume density of the testicular interstitium. No differences were observed between the groups in the proportion of proliferating and apoptotic cells. Finally, undernourished animals exhibited higher concentrations of testosterone in blood serum (CG 2,90 ± 1,51* vs RG 5,25 ± 3,53), and their myoid cells demonstrated increased expression of androgen receptors. In conclusion, our findings suggest that undernutrition during early life stages fetally programs the rat, negatively affecting its body development, altering testosterone production and testicular histophysiology in adults older than one year of age.

## Introduction

Maternal malnutrition during critical periods of offspring dependency is responsible for determining permanent effects on postnatal growth and development. This effect in which the uterus’ environment is influential in the offspring’s health later in life is referred to as “fetal programming” (Desai and Hales, 1997). The fetus’ response to this nutrient or oxygen deficiency is a reduction in its mitotic rate. The faster the tissues divide, the more evident this effect is (Cooper et al., 2002). As far as Sertoli cells are concerned, their number per testis is highly correlated to daily sperm production since each of them is limited in its capacity to provide metabolic support to germ cells (Russell and Peterson, 1984; Orth et al., 1988). Specifically in rats, the first of these cells emerges around gestation day 13.5 (Magre and Jost, 1991), normally proliferating during fetal life. Between day 16 (Steinberger and Steinberger, 1971) and 17.5 (Angelopoulou et al., 2008) after birth Sertoli cells (SC) cease their proliferation. Regarding their functions, the regulation of myoid cell (MC) number through trophic factors and the promotion of the development of adult Leydig cells (LC) from their precursor cells in the prepubertal stage (Rebourcet et al., 2014a), are extensively described. An equally relevant function is their role in maintaining the LC and MC number as well as their activity in the adult testis (Rebourcet et al., 2014b; Wen et al., 2016). The normal functioning of the aforementioned cells is influenced by several factors, among which androgen presence is of great relevance (Escott et al., 2014). Androgen receptor (AR) expression in SC is initiated very early in life, more precisely since fetal stages. Its absence has negative consequences, such as a reduction of SC numbers in each testis and a deficiency in their ability to support germ cells (Tan et al., 2005). For normal spermatogenesis to happen and subsequent fertility (Chang et al., 2013), AR presence in SC is crucial, even from fetal stages (Rey et al., 2009). The testis which is both, an exocrine and endocrine gland in nature, houses LC, which start secreting androgens since fetal age (Smith and Walker, 2014). Specific AR in target cells is required in order for this hormone to play its role. Androgen presence is the main regulator, through paracrine mechanisms, of AR expression in the testis (Zhu et al., 2000a). It is broadly understood that both MC and SC, must express AR for gametogenesis to take place (O'Hara and Smith, 2015). In response to androgens, LC stimulate themselves resulting in an increase of AR expression through autocrine positive feedback (Wang et al., 2009; Griffin et al., 2010). Apoptosis progresses into an irreversible stage once caspase 3 is activated (Ola et al., 2011). Our previous findings indicate that nutritionally restricted males during gestation and lactation exhibit reduced body and testicular weight at 100 days of postnatal life, with a lower number of SC per cross-section of the seminiferous tubule and total per testis (Genovese et al., 2010). Additionally, these males show a higher proportion of apoptotic MC as compared to controls (Genovese et al., 2019). Lastly, a one year old male rat is considered middle-aged (Hussein et al., 2020). To the best of our knowledge, no studies have demonstrated programming effects on testicular histophysiology resulting from subnutrition during gestation and lactation in the long term. Our hypothesis was that subnutrition during gestation and lactation affects body development and testicular histophysiology in the long term. Accordingly, the aim of this study was to describe the effects of subnutrition during gestation and lactation on body and testicular development, as well as testicular function, in one- year- old mature adult rats.

## Methods

All procedures involving animals were in compliance with the following national law:18.611 (https://www.cnea.gub.uy). The evaluation and approval of the experimental protocol: 573/17 was responsibility of the Ethics Committee in the use of animals (CEUA) from Universidad de la República, Uruguay. No animals were excluded from the experiment. The use of predefined endpoint criteria was not necessary.

### Preliminary experiment

To determine *ad libitum* food intake for every day along the rat’s pregnancy, six primiparous female Wistar rats, approximately the same age (3 months old) as the ones in the definitive experiment, all mated by the same male rat, were used. The environmental conditions were the same as those used later (in the definitive experiment), including who handled the animals and the food. After effectively determining pregnancy, body weight and food intake were registered daily at the same time along the rats’ pregnancy.

The average *ad libitum* food intake for every day along the rats’ pregnancy was estimated from this preliminary experiment. Afterwards, this value was reduced to 50%, which was the daily food intake along the rats’ pregnancy of the treated group in the main experiment.

### Main experiment

The experiment encompassed gestational food restriction of the offspring by restricting their mothers’ food intake during the whole pregnancy as well as postnatal food restriction during the lactation period by increasing the number of pups per litter. It took place in the Animal Experimentation Laboratory (LEA) of the Veterinary Faculty from Universidad de la República, Uruguay. Wistar rats were housed in a room illuminated for 12 hours, with 50% humidity and 22°C temperature. Balanced composition, standard rat chow (23% protein; 8% fibre; 2.5% calcium; 1.25 phosphorus) was always used.

At the beginning of the experiment, thirty primiparous female 3 month old Wistar rats (229.7 ± 8.4g body weight), were all mated by the same male (initial body weight: 386g; final body weight after 17 days: 407g). They were randomly distributed into one of the two experimental groups described below.

### Pregnancy period of the experiment

Pregnancy was confirmed by a daily vaginal smear (Marcondes et al., 2002). It involved placing and promptly removing 100 microliters of intra-vaginal physiological saline solution with a dropper. The resulting liquid was spread on a slide and examined under a microscope. Presence of spermatozoa in the smear was taken as day one. Pregnant females were organised into the following experimental groups:

**Control Group (CG**, n=15**):** mothers fed during pregnancy with *ad libitum* access to food and water.

**Restricted group (RG,** n=15**):** pregnant rats were nutritionally deprived during pregnancy by restricting their food intake at 50% of the maternal daily *ad libitum* volume estimated in the preliminary experiment. Consumption was daily adjusted during pregnancy with the consumption record of the animals already assigned to the control group.

All pregnant females from the two experimental groups were housed in individual cages. Maternal body weight was daily registered. Prior to the start of the experiment, the end-point criterion for treated animals was established as a loss of more than 10% of their initial body weight. All mothers were weighed 6 hours after parturition CG= 270.4 ± 14.5 and RG = 233.9 ± 12.6 resulting in an average weight gain of 2.8 ± 10.3 grams in the females of the RG. The number of pups born per mother was counted.

### Lactation period of the experiment

From parturition (day 22 of the experiment) both groups of mothers were offered *ad libitum* access to food and water. Litters in group C consisted of 8 pups until weaning. Litters in the restricted group (RG) consisted instead of 14 pups during lactation (given the rats have 12 nipples and 14 pups per litter, the amount of milk available to each pup was reduced). At parturition, litter size was monitored and, when necessary, pups were removed from or added to donor dams to maintain a fixed number of suckling offspring according to the experimental group. All donor animals were of the same age and were marked on the dorsal skin using a permanent marker. The proportion of male and female pups was maintained at 50% each in all litters. Pups born to the mother corresponding to each treatment were individually identified by ear notching in their pinna at 20 days of life.

### Adult growth period

At postweaning (25 days of life), one male offspring per mother was randomly selected (RG n=15; CG n=15). Animals were housed in groups of 5 per cage, with the same cage mates maintained till day 365 of life, and with *ad libitum* access to food and water.

### Sampling and histological processing

On day 365 of postnatal life all animals were weighed, measured from their nose to their tail tip, anesthetised with Xilacine (20mg/kg) and Ketamine (60mg/kg), and decapitated with a guillotine. Blood without anticoagulant was collected, then centrifuged to separate the serum, which was stored at -20 °C until assayed.

Both right and left testes were dissected, weighed and immersion-fixed in Bouin’s solution during 12 hours. Testes were stored in ethanol 70º (v/v), immersed in a series of increasingly concentrated ethanol baths, impregnated in chloroform and finally in liquid paraffin to make blocks that were then cut into 5 µm thick sections.

Images were captured (light microscope Olympus BX50, video camera Infinity 1; Olympus and Sony, Tokyo, Japan, respectively and Image Pro Plus Media Cybernetics program, Silver Spring, MA, USA) at a final magnification of 2500x in the computer’s screen from slides treated with Hematoxylin and Eosin, and one section per testis per animal was analysed. Testicular volume was estimated from testicular weight, assuming that the testicular density is 1 (Mori and Christensen, 1980; Johnson et al., 1981). The volume density of seminiferous tubules and testicular interstitium was determined by point counting, superimposing a grid of 100 points in 30 randomly taken images of each animal’s testicular parenchyma. The points that fell on each histological structure of interest were counted. The volume density (Vv) of such structure was calculated as follows:

**Vv = Pn/Pt**, where Pn is the number of points of the grid that fell on the given structure and Pt is the total number of points per image.

The seminiferous tubules’ diameter was determined by measuring two perpendicular diameters in 50 seminiferous tubule transverse cross-sections.

The total number of SC per cross section of seminiferous tubules was counted in 50 cross sections.

The total number per testicle of SC was calculated with the following equation:

Total number of cells = Ns x (L/thickness of the histological section), where Ns is the average number of nuclei per seminiferous tubule cross-section and L is the total length of the seminiferous tubules of a given testicle.

The seminiferous tubules were assumed as cylindrical and their lengths were estimated from the following equations (Marshall and Plant, 1996):

L = Vs/(π[Ds/2]^2^), and Vs = (Vv seminiferous tubules) x absolute testicular vol, where Vs is the seminiferous tubules total volume and Ds is the diameter of these tubules. The cell number per seminiferous tubule cross section was multiplied by the seminiferous tubules total length in order to obtain the number of cells per testicle. Interstitial testicular volume was obtained by subtracting the absolute volume of the seminiferous tubules and artifacts.

### Immunohistochemistry (IHC)

#### Androgen receptor (AR)

**Figure 1:**
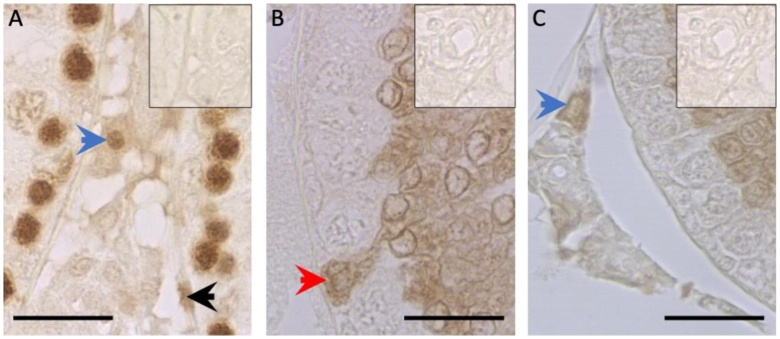
PCNA (proliferating cells) or Caspase-3 (apoptotic cells) expression in testis from one-year-old-animals. A: PCNA staining, positive LC (blue arrow), positive MC (black arrow). B and C: Caspase-3 staining. B: positive SC (red arrow) and C: positive LC (blue arrow). Squares present at the top-right corner in the images A-C correspond to the negative control in the run. Scale bar: 50 µm.

Slides were treated with a mouse anti-human AR primary antibody (AR N-20 SC-816, Santa Cruz, USA) and an amplifier kit Mach 2 Double (Mouse-HRP + Rabbit-AP, Polymerdetection kit, Biocare medical, USA) with DAB chromogen, and one section per testis per animal was analysed. A slide without primary antibody was used as the negative control. The AR relative abundance in SC, LC and MC was described as relative intensity. 300 cells of each type were evaluated using a semi-quantitative intensity scoring (IC) 0-3 scale, where 0 = negative, 1=weak, 2=moderate and 3=strong positivity. Then, a positivity index (PI) for each cell type and for each animal was calculated as follows (Figure 1):

PI =1 x n (IC1) + 2 x n (IC2) + 3 x n (IC3), where n = number of cells exhibiting IC = 1, 2 o 3, expressed in % (Boos et al., 1996).

#### Proliferating cells (PCNA) and apoptotic cells (caspase 3)

Primary antibody anti-caspase 3 (AB 4051, Abcam, UK) or anti-PCNA (Dako M0879, USA) was used on different slides obtained from each animal. Specifically, one section per testis per animal was analysed. The amplifier kit used was the same as for the AR. The percentage of SC, LC and MC positive for each antigen in 300 cells was estimated in randomly selected cross sections of seminiferous tubules and its interstitial spaces.

### Measurement of testosterone in blood serum

Serum samples from each animal in each group were assayed in the Animal Laboratory of Endocrinology and Metabolism, Veterinary Faculty, Montevideo, Uruguay. Testosterone concentrations were determined by a direct solid-phase radioimmunoassay (RIA) using CISBIO kits (TESTO-CT2, CISBIO, France). The sensitivity of the assay was 0.148 nmol/L. The intra-assay CV for control 1 (4.00 nmol/L) was 7.99%.

### Statistical analysis

The results for all variables were analysed using the Shapiro- Wilk test to assess normality. All quantitative variables were expressed as mean ± sd and T tests were used to examine them. The differences among groups for the quantitative histological variables including both, positivity index and testicular weight were compared by T tests. Group effect and individual effect within group were studied and considered different if p≤0.05. Post hoc differences among groups were also studied with Tukey tests. Pearson correlations were studied among the different variables, specifically between testicular weight and the different immunohistochemical variables, as well as serum testosterone concentration. The software used for the mentioned analysis was Statistica version 6 (Palo Alto CA, U.S.A).

## Results

The treatment negatively affected the average number of pups born (CG= 14.7 ± 2.1 vs 12.9 ± 1.9 p=0.019). Body weight at birth was lower in the RG animals (CG= 6.9 ± 0.4 vs 6.3 ± 0.3 p=0.001). Throughout the postpartum period and up to one year of life, no male showed signs of clinical illness.

All variables were normally distributed. In general terms, we observed that animals from the RG were different as compared to the CG in almost all the variables studied. At one year of life, RG animals showed lower body weight, body length and testicular weight (Table 1).

**Table 1:** Macroscopic variables.

Group	BodyWeight (g)	Body Length (cm)	Testicular Weight (g)
Control	592.9 ± 33.4***	47.2 ± 0.7 ***	1.82 ± 0.05 ***
Restricted	527.5 ± 23.5	45.1 ± 1.6	1.69 ± 0.06

Means ± sd. ***P<0.001 compared to the control group

### Morphometrical variables

We found a decrease in the absolute volume and diameters of the seminiferous tubules, as well as in the number of SC both per tubule section and total per testis in the RG animals in relation to the CG (Table 2).

**Table 2:** Morphometrical variables.

Group	AVST (mL)	DST(µm)	SCSN	SCTN
Control	1.31 ± 0.05**	222.2 ± 14.2	23.4 ± 0.32***	134,6 ± 24,9 x 10^4^*
Restricted	1.21 ± 0.01	221.8 ± 9.6	21.8 ± 0.34	111,4 ± 14,4 x 10^4^

Absolute volume of seminiferous tubules (AVST), diameters of seminiferous tubules (DST), SC per tubule section (SCSN) and total SC number per testis (SCTN) from control animals (CG) and nutritionally restricted throughout gestation and lactation (RG) at 1 year of life (means ± sd). *P≤0.05; **0.001≤P<0.05; ***P<0.001 compared to the control group.

### Testosterone serum concentration

We found that testosterone serum concentration was higher in the RG 5.25 ± 3.53 vs CG 2.90 ± 1.51* (means ± sd; ^*^: P≤0.05).

### IHC-based studied variables

#### Celular proliferation (PCNA positive cells) and apoptosis (Caspase 3 positive cells)

None of the cells studied for detection of PCNA or Caspase-3 by IHC- techniques showed differences between RG and CG at one year of life (Table 3, Figure 1).

**Table 3:** Percentage of immunostained cells for each marker PCNA and Caspase-3.

	CG	RG
% PCNA SC	-	-
% PCNA LC	85.8 ± 9.6	76.5 ± 19.2
% PCNA MC	95.0 ± 2.4	90.0 ± 6.3
% Cas SC	2.2 ± 0.8	1.9 ± 1.0
% Cas LC	8.6 ± 2.6	8.3 ± 2.2
% Cas MC	1.1 ± 0.8	0.8 ± 0.6

Percentage (%) of immunostained cells for each marker (PCNA and Caspase-3) in SC, LC and MC from the CG and RG’s testis 1 year of life (means ± sd).

### Androgen receptor

**Figure 2:**
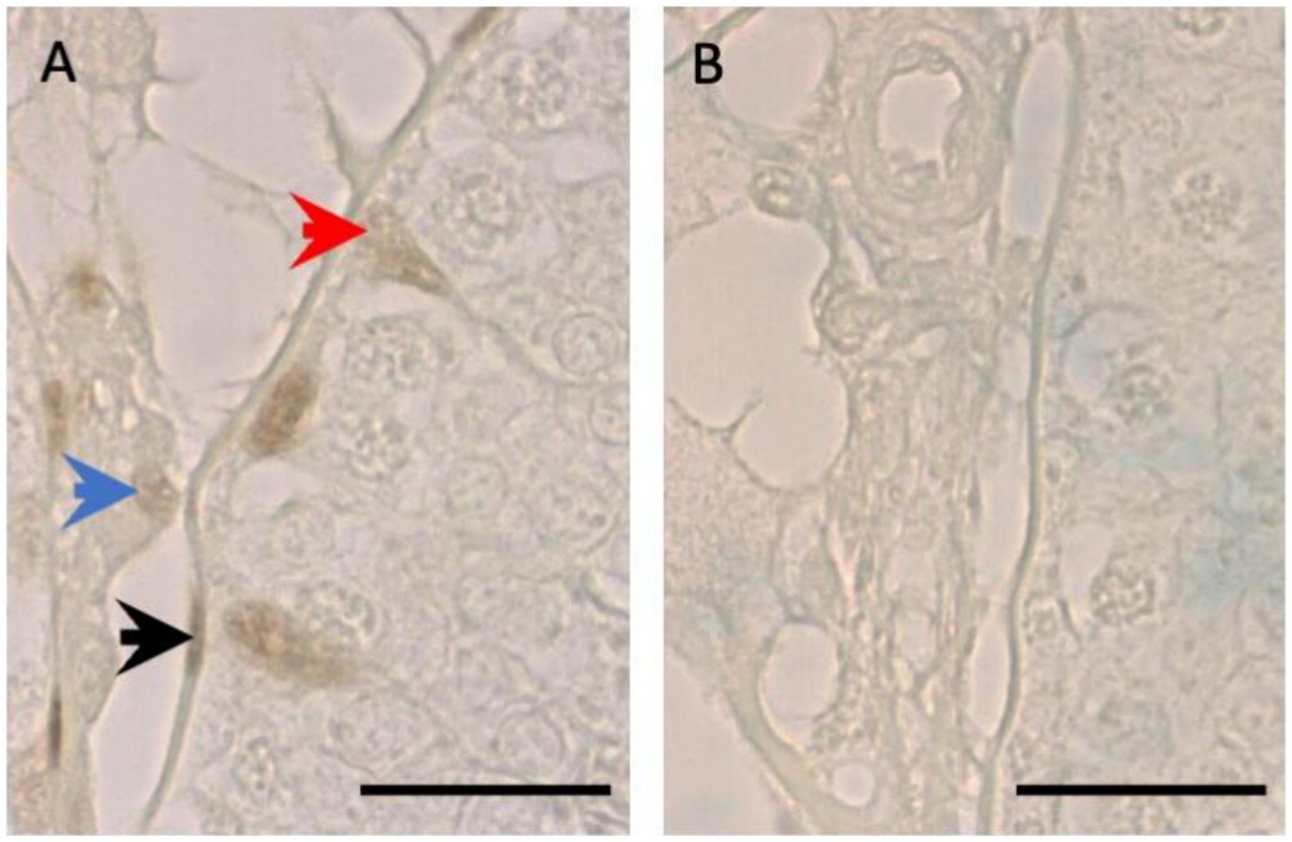
AR expression in testes from one-year-old animals. A: AR staining, positive SC (red arrow, representing 2 in the IC scoring system), positive LC (blue arrow, representing 1 in the IC scoring system) and MC (black arrow, representing 3 in the IC scoring system). B: negative control in the run. Scale bar: 50 µm. In B, all nuclei representing 0 in the IC scoring system are observed.

The AR positivity index in MC was higher in the RG than in the CG. No differences were found between groups in the other studied cells (Table 4, Figure 2).

**Table 4:** Positivity index of immunostained cells for AR.

Group	ARPI SC	ARPI LC	ARPI MC
Control	0.8 ± 0.4	0.9 ± 0.2	1.3 ± 0.2*
Restricted	0.8 ± 0.2	0.9 ± 0.4	1.7 ± 0.3

Positivity index (PI) of immunostained cells for AR in SC, LC and MC at 1 year of life (means ± sd). *P≤0.05 compared to the control group.

### Correlations

A correlation was found between body weight and body length (0.77 p=0.043) and testicular weight with TNSC (0.80 p=0.029). The remaining variables studied showed no correlations.

## Discussion

To our knowledge, this is the first study that examines the effects of subnutrition during gestation and until 25 days of life in 1-year-old mature adult animals. We found that it negatively affects body weight and length, testicular weight and testicular morphology, with a lower number of SC per cross-section of seminiferous tubules and total per testis; it also modifies AR abundance in MC and increases serum testosterone concentration.

Several reports demonstrate negative effects of subnutrition throughout gestation on body development and testicular parenchyma when studying newborn animals (Genovese et al., 2011); these effects are also found when studying young adults who were underfed during both gestation and lactation (Genovese et al., 2010; Genovese et al., 2019; Pedrana et al., 2020; Genovese et al., 2024). We have also found that the treatment negatively affects the animals’ body length, from their nose to their tailtip. However, a treatment with half the casein in the maternal diet while gestation takes place, provokes an increase in the males’ body weight at 850 days postpartum (Rodriguez-Gonzalez et al., 2014). We understand that the differences in the results could be due to both, the age at which the samples were taken and the different nutritional treatments. In our work the animals were nutritionally restricted by giving them 50% of the *ad libitum* food consumption. Apparently, this did not affect them in the same way as would a decrease in the proportion of the protein intake by the pregnant mother. In our experimental design we cannot differentiate the effect of treatment during gestation from the effect of treatment during lactation on the final outcome. Recently, we determined that subnutrition applied only during the second half of gestation suffices for animals to have lower body and testicular weights at puberty (Genovese et al., 2024). Therefore, more studies are needed to differentiate the long-term effects of treatment during pregnancy from those during lactation.

Our work demonstrates that subnutrition during pregnancy and lactation negatively affects body weight at one year of age or, in other words, 340 days after having finished treatment. Therefore, such effects would be irreversible, or at least very long lasting. In addition, our treatment also causes a decrease in testicular weight, an effect that has been previously described for different ages (Genovese et al., 2019). Newborns have lower testicular weight when their mothers are undernourished during gestation (Genovese et al., 2011). Such difference is maintained in 100-day-old animals that were nutritionally restricted during gestation until 25 days of life (Genovese et al., 2010). Even subnutrition in the second half of gestation causes lower testicular weight when compared to controls at puberty at 40 days of life (Genovese et al., 2024). At one year of age, testicular weight is still lower. This indicates that the treatment effect (subnutrition with 50% of maternal consumption during pregnancy and the increase in the litter size during the lactation period) continues at least 340 days after having finished the treatment.

### Morphometry results

We found a lower number of SC both per cross section of seminiferous tubules and total per testis in RG animals. For a better interpretation of this result it has to be taken into account that a strong correlation exists between both SC number and adult testicular size in a wide variety of mammals including the rat (Sharpe et al., 2003) and between SC number and daily sperm production (Russell and Peterson, 1984; Berndtson and Thompson, 1990). This is of utmost importance due to the limited capability of SC of metabolically supporting germ cells (Orth et al., 1988; da Silva et al., 2006). We had already found a similar result in young adult animals at 100 days of life (Genovese et al., 2010). Therefore, the present work indicates that RG animals continue with a diminished capacity of theoretical maximal sperm production at one year of life, representing a negative effect on reproductive performance.

### Apoptosis / proliferation

We found no differences in the proportion of SC undergoing apoptosis among the experimental groups. We expected that the lower number of SC per seminiferous tubule cross section at 365 days would be explained by the higher proportion of SC undergoing apoptosis (i.e., positive for activated caspase 3). We deduce that the lower number of SC could be accounted for either by an increase in cell death at an earlier stage or by a decrease in proliferation during the period when these cells are multiplying.

We found no differences in the proportions of apoptotic LC or MC. In this respect, we previously found in a similar experiment using the same immunohistochemistry technique, an increase in the proportion of apoptotic MC in the RG at 100 days of life (Genovese et al., 2019). This might be explained if we take into account that Wistar males reach sexual maturity at 90 to 100 days of age (Robb et al., 1978). Therefore, until approximately 100 days of life the animal is increasing testicular development and reorganising its cytoarchitecture, which could be altered by the treatment. At one year of age, on the other hand, it has been stable for several months and this could explain why MC no longer expresses the aforementioned difference.

As far as PCNA expression is concerned, no effect of treatment on the proliferation proportion of MC, LC and SC has been found. Since LC preserve their ability to divide throughout life (Stanley et al., 2012), they can maintain their number adjusted to the current testicular histophysiological need. MC also have the ability to multiply throughout life. However, the cells responsible for maintaining the number of MC and their activity in the testis are SC (Wen et al., 2016). Thus, it could be assumed that SC capacity to stimulate MC proliferation was not affected by the treatment. In reference to SC proliferation in the rat, it has to be considered that it peaks on gestation day 20 (Yang et al., 1990) and normally stops at about 16 (Steinberger and Steinberger, 1971) -17.5 days after birth (Angelopoulou et al., 2008). As such, there are no PCNA immunoreactive SC at 1 year of age. Having said that, it might happen that the difference in the SC number per seminiferous tubule cross section can be accounted for a decline in the mitotic rate in early life stages. In this connection, we have studied the proportion of PCNA positive SC in a similar design at 2 days of life and no differences were found (Genovese et al., 2011). Therefore, further studies that go in depth into the treatment effects on the SC proliferation at ages when they are still dividing are needed.

### Serum testosterone concentration and androgen receptor

#### Testosterone concentration

We found an increase in blood serum testosterone concentration in the RG. In order to interpret this result, it is important to consider that the impact of different pathologies on the hypothalamic-pituitary-gonadal axis and both, testosterone production and its blood concentration has been studied for decades. In connection with this, it is widely known that bilateral cryptorchidism in adult rats causes increase in LH, FSH and testosterone (Gomes and Jain, 1976). Other researchers observed (also in adult male rats) that hemicastration provokes a compensatory increase in testosterone secretion, independently of the FSH and LH blood concentrations (Brown et al., 1991). Furthermore, the increment in blood testosterone concentration would be linked to the innervation of the surviving contralateral testis (Zhu et al., 2000b). It was subsequently observed that orchiectomy could lead to compensatory hypertrophy of the contralateral testis, resulting in a similar testosterone production to that of intact rats (Wen et al., 2013). In addition to this, it is a well-known fact that male mature rats (14 months) produce less testosterone than at 3 and 6 months of age, which is considered a normal physiological issue (Mendis-Handagama and Ariyaratne, 2001). Hence, older animals are expected to produce less testosterone. From this background knowledge it follows that the testicular parenchyma attempts to compensate for some pathologies (monorchidism and hemi-castration) by producing more testosterone. Thus, we assume the increase in hormone concentration in the RG in our experiment corresponds to the testicles’ response to compensate a similar damage, at least in some aspects, to orchitis or hemicastration.

When it comes to morphologically explaining the changes that justify the androgen increase, we find conflicting literature sources. On the one hand, it has been found in rats that the increase in blood testosterone is not linked to compensatory testicular hypertrophy (Lindgren et al., 1976), while on the other hand other researchers describe an increase in the LC number with interstitial hypertrophy (Agee et al., 1988). It has also been demonstrated using the rabbit as a model, that prepubertal hemicastration led to compensatory hypertrophy in the contralateral testis when puberty was reached (Sanefuji, 1988). More recently, it has been proved that hemicastration produces compensatory hypertrophy of the remaining testis and that this hypertrophy decreases as the animals age, without a reduction in serum testosterone levels (Ahmadnia et al., 2019). We found no treatment effect on the volume density of the testicular interstitium. However, our experiment was neither designed to study the effect of treatment specifically on the interstitium nor on the hypothalamic-pituitary axis that controls it. It is unfortunate our work did not enable the measurement of FSH and LH concentrations.

In addition, recent studies have demonstrated a relationship between the gut microbiota and reproduction (Ashonibare et al., 2024), and have even established a positive correlation between certain microorganisms and blood testosterone concentration (Haro et al., 2016). In rats, a link has been reported between the maternal microbiota and the development of obesity in the offspring (Paul et al., 2016), increased blood insulin levels, hippocampal functions, and even circulating thyroid hormone concentration (Forgie et al., 2020). More recently, it has been shown that an abnormal imbalance of the male gut microbiota may have a maternal origin in early life stages and be expressed, among other outcomes, as infertility in humans (Lv et al., 2024), as well as affecting the age at puberty (You et al., 2025). In all these studies, the experimental treatments were based on imbalances in some specific dietary components, in greater or lesser proportion. In contrast, in our study we restricted the amount of food consumed by treated females during gestation while administering a standard rat chow with a balanced composition; thus, the proportion of nutrients was not altered. We understand that our results may be modulated by the gut microbiota. However, to our knowledge, no studies have yet linked testicular morphology, relative abundance of AR, testosterone concentration, and the gut microbiota of adult males with the maternal gut microbiota in our conditions. Therefore, we acknowledge that further studies are necessary to understand the origin of the increase in testosterone and the variation in the concentration of these hormones throughout life.

### Androgen receptor

Having analysed androgen receptor PI we found that MC of the RG were the only cells affected by the treatment and they express more AR than the same cells in the CG.

It is well established that AR production is stimulated by the presence of androgens produced by LC (Wang et al., 2009). These receptors are essential for the testis to perform its normal gametogenic function, and they must be especially present in the CS and MC (O'Hara and Smith, 2015). In this regard, it has been established that SC exhibit relatively stable AR expression throughout life, whereas LC demonstrate a peak in AR expression during puberty, which subsequently stabilizes in adulthood (Abd El-Meseeh et al., 2016). In a comparable experiment and employing identical immunohistochemical techniques, animals subjected to nutritional restriction during gestation until 25 days of life, presented the same AR expression as their controls in both SC and LC at 2, 25 and 100 days of life. However, a lower AR expression in MC at 2 and 25 days of life in the RG was displayed (Genovese et al., 2019). The literature indicates that the MC can modulate the expression of this receptor throughout life, particularly under adverse nutritional conditions during early developmental stages. In order to interpret the processes occurring within these cells, it is essential to recognise that SC play a critical role in maintaining the population of MC by stimulating their differentiation from precursor cells. Furthermore, SC are also involved in promoting MC normal functioning (Rebourcet et al., 2014a; Wen et al., 2016). We observed that, in the presence of higher testosterone concentrations, SC express AR normally, that is, at levels comparable to those of the controls. In contrast, MC, which are dependent on SC, do not behave in the same way. Consequently, it is plausible that the treatment modifies the sensitivity of MC to testosterone or that we are observing a symptom of undetected failures in SC regarding their functional regulation of MC.

### Correlations

The studied correlations largely confirmed the findings that have been established for decades between variables such as body weight and body length or testicular weight and number of SC. We did not find any correlations between the variables studied by IHC and the macroscopic and/or morphometric variables.

## Conclusion

In conclusion, under our experimental conditions the long term effects of fetal programming due to subnutrition in early stages predominantly lead to lower body and testicular weight, lower number of SC, higher concentrations of testosterone in blood serum and higher expression of AR in MC. Therefore, fetal programming in early stages of life affects the morphology and testicular function of the mature adult male rat.

## Data Availability

Research data is available in the body of the article.
